# Selective tract abnormality in adrenoleukodystrophy: Uncommon MRI finding

**DOI:** 10.4103/0972-2327.40230

**Published:** 2008

**Authors:** Rohit Bhatia, Soaham Desai, M. V. Padma, Manjari Tripathi, Kameshwar Prasad

**Affiliations:** Department of Neurology, All India Institute of Medical Sciences, New Delhi - 110 029, India

**Figure 1:**
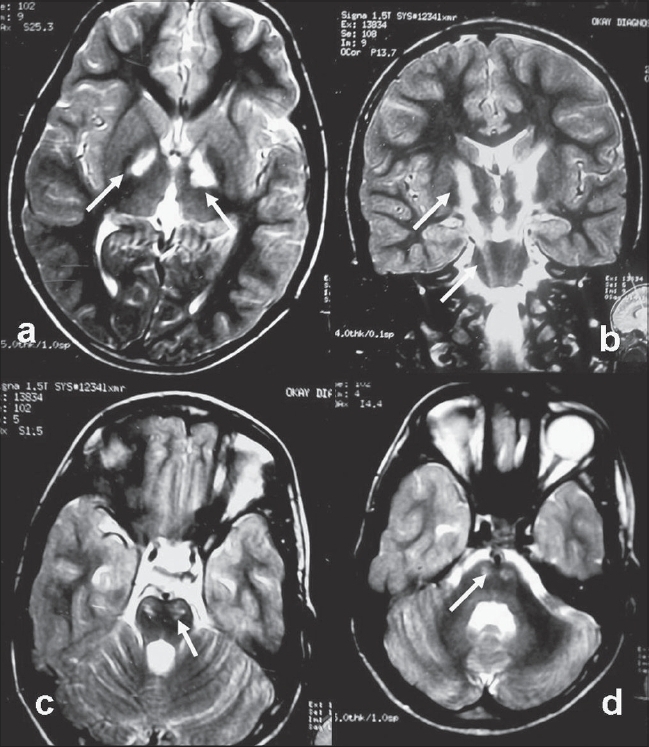
MRI Brain T2W images showing symmetrical hyperintensity (indicated by arrows) along the bilateral descending tracts in the internal capsule (axial image (a) and coronal image (b) and through the brain stem in the pons (c) and medulla (d), with associated brain stem atrophy

A 9-year-old male child presented with progressive spastic pure motor quadriparesis of one year duration without any visual, auditory or extrapyramidal dysfunction. Mild cognitive impairment was present. His hemogram, liver, renal, thyroid and parathyroid profile, serum cortisol and serum ACTH were normal. His MRI brain revealed symmetric T2W hyperintensity along the descending tracts in the genu [Figure 1A], the posterior limb of internal capsule and along the brainstem [Figure 1B–D]. His MRI of spine was normal. Elevated levels of very long chain fatty acids (VLCFA) in serum confirmed the diagnosis of childhood cerebral X-linked adrenoleukodystrophy (ALD).

X-linked ALD is a peroxisomal disorder that affects the white matter of the central nervous system, adrenal cortex and testes and is caused by a deficiency of a single enzyme, acyl-CoA synthesase, that prevents the breakdown of very long chain fatty acids (C > 22:0); which (VLCFA) subsequently accumulate in the tissues and plasma, thereby leading to cerebral demyelination, peripheral nerve abnormalities and adrenocortical and testicular insufficiency. The genetic defect responsible for X-linked ALD is located in Xq28, the terminal segment of the long arm of the X chromosome. Loes *et al.* described five MR imaging patterns, with their relative frequencies, ages of affected patients and patterns of progression at MR imaging.[1] The MRI in adrenoleukodystrophy classically shows the primary involvement of the deep white matter in the parieto-occipital lobes and involvement of the splenium of the corpus callosum (66% of cases are mainly found in children.), which may include the lesions of the visual and auditory pathways. The involvement of the frontal lobe or genu of the corpus callosum is observed in approximately 15.5% of cases and mainly in adolescents. Primary cerebellar white matter or combined involvement of the parieto-occipital and frontal white matter has also been described. The primary involvement of the frontopontine or corticospinal projection fibers, as seen in our case, is reported in 12% of the cases, mainly in adults. However, it may also be observed with childhood cerebral ALD, as in the present case.[2,3]
